# Limitations of antifungal prophylaxis in preventing invasive *Candida* surgical site infections after liver transplant surgery

**DOI:** 10.1128/aac.01279-23

**Published:** 2024-02-01

**Authors:** M. Carugati, S. Arif, M. E. Yarrington, L. Y. King, M. Harris, K. Evans, A. S. Barbas, D. L. Sudan, J. R. Perfect, R. A. Miller, B. D. Alexander

**Affiliations:** 1 Department of Medicine, Division of Infectious Diseases, Duke University, Durham, North Carolina, USA; 2 Department of Medicine, Division of Gastroenterology, Duke University, Durham, North Carolina, USA; 3 Department of Pharmacy, Duke University, Durham, North Carolina, USA; 4 Department of Surgery, Division of Abdominal Transplant Surgery, Duke University, Durham, North Carolina, USA; University Children's Hospital Münster, Münster, Germany

**Keywords:** micafungin, prophylaxis, transplant, liver

## Abstract

Invasive primary *Candida* surgical site infections (IP-SSIs) are a common complication of liver transplantation, and targeted antifungal prophylaxis is an efficient strategy to limit their occurrence. We performed a retrospective single-center cohort study among adult single liver transplant recipients at Duke University Hospital in the period between 1 January 2015 and 31 December 2020. The study aimed to determine the rate of *Candida* IP-SSI according to the peri-transplant antifungal prophylaxis received. Of 470 adult single liver transplant recipients, 53 (11.3%) received micafungin prophylaxis, 100 (21.3%) received fluconazole prophylaxis, and 317 (67.4%) did not receive systemic antifungal prophylaxis in the peri-transplant period. Ten *Candida* IP-SSIs occurred among 5 of 53 (9.4%) micafungin recipients, 1 of 100 (1.0%) fluconazole recipients, and 4 of 317 (1.3%) recipients who did not receive antifungal prophylaxis. Our study highlights the limitations of antifungal prophylaxis in preventing invasive *Candida* IP-SSI after liver transplant surgery. We hypothesize that pathogen, host, and pharmacokinetic-related factors contributed to the occurrence of *Candida* IP-SSI despite antifungal prophylaxis. Our study reinforces the need for a risk-based, multi-pronged approach to fungal prevention, including targeted antifungal administration in patients with risks for invasive candidiasis and close monitoring, especially among patients with surgically complex procedures, with timely control of surgical leaks.

## INTRODUCTION

As *Candida* is part of the gastrointestinal microbiome, it can translocate into the peritoneal cavity at the time of liver transplantation and cause invasive surgical site infections. This contributes to the high rate of invasive *Candida* infections reported in the liver transplant population and the need for antifungal prophylaxis at the time of surgery for liver transplant candidates at an increased risk for invasive *Candida* infections (1, 2). The American Society of Transplantation (AST) recognizes re-transplantation, re-operation, renal failure requiring hemodialysis, transfusion of ≥40 units of cellular blood products, choledochojejunostomy, and *Candida* colonization in the perioperative period as risk factors for *Candida* infection among liver transplant recipients and recommends antifungal prophylaxis for 2–4 weeks following transplantation in this setting (2). While no final recommendation regarding the preferred antifungal agent in high-risk liver transplant recipients is provided, the AST guidelines suggest similar performance of echinocandins and fluconazole in the prevention of *Candida* infections after liver transplant surgery (2). Of note, however, more recent studies questioned the efficacy of echinocandin prophylaxis after transplant surgery, given concerns about the tissue penetration of echinocandins, particularly into the pleural space (3). In this study, we assessed the epidemiology and outcomes of invasive *Candida* surgical site infections among adult liver transplant recipients according to the peri-transplant antifungal prophylaxis received over a 6-year period at a major US transplant center.

## MATERIALS AND METHODS

### Study design

We performed an observational single-center retrospective cohort study of all adult patients who underwent a single liver transplant between 1 January 2015 and 31 December 2020 at Duke University Hospital (Durham, North Carolina), a high-volume solid organ transplant center that has been performing adult and pediatric solid organ transplants since 1965. This study was approved by the Duke University Health System Institutional Review Board (IRB number: Pro00104142). Inform consent of study participants was waived by the IRB.

### Study population

Eligible patients were 18 years of age or older and met all the following criteria: (i) single-liver transplant performed at Duke University Hospital during the 6-year study period and (ii) at least 12-month post-transplant clinical follow-up available, unless death occurred before the 12-month mark.

### Definitions and adjudication process for invasive primary surgical site infections

Using the Centers for Disease Control and Prevention—National Healthcare Safety Network definitions for surgical site infections, surgical site infections needed to occur within 3 months after the transplant procedure (4). Deep incisional and organ/space infections involving the primary surgical incision were considered invasive primary surgical site infections (IP-SSIs). Additional details on the adjudication process for IP-SSI are described in our previous publication (5).

### Other study definitions

Antifungal prophylaxis was defined as any systemic prophylactic antifungal administered within 24 hours of surgical incision. Antifungal prophylaxis regimens were categorized into three groups: micafungin prophylaxis, fluconazole prophylaxis, and no antifungal prophylaxis. The institutional antifungal prophylaxis protocol is reported in Table 1: while fluconazole was the recommended antifungal agent, micafungin prophylaxis was used in the case of prior infections with non-*albicans* isolates, prior prolonged azole exposure, or contraindications to use fluconazole (including prolonged QTc or prior hypersensitivity reaction). The duration of antifungal prophylaxis was calculated in days as interval from the time of transplant until the discontinuation of prophylactic antifungal agent. For those patients receiving antifungal prophylaxis at the time of *Candida* IP-SSI diagnosis, the duration of antifungal prophylaxis was calculated in days as the interval from the time of transplant until the diagnosis of *Candida* IP-SSI. *Candida* antifungal susceptibility testing was performed by the Duke Microbiology Laboratory in accordance with the Clinical and Laboratory Standards Institute (CLSI) methods, breakpoints, and interpretative categories. Length of hospital stay was defined as the number of days from the admission date to the date of discharge during the index transplant hospitalization. In-hospital mortality was defined as all-cause mortality during the index transplant hospitalization. One-year mortality was defined as all-cause mortality from the time of transplant to 365 days after transplant (5).

**TABLE 1:** Perioperative antifungal (excluding *Pneumocystis jiroveci*) prophylaxis protocol for adult liver transplant surgery at Duke University Hospital in the period between 1 January 2015 and 31 December 2020^*a*^

Antifungal prophylaxis protocol
Standard
Clotrimazole 10 mg q12h (for 3 months)
Fulminant liver failure
Fluconazole 400 mg q24h^*b*^ assuming estimated creatinine clearance >50 mL/min (for 5 days or as clinically indicated)
Split liver
Fluconazole 400 mg q24h assuming estimated creatinine clearance >50 mL/min (for 2 days or as clinically indicated)
History of *Candida* infection in donor or recipient
Individualized antifungal prophylaxis

^*a*^ Starting in 2018, in the case of intraoperative transfusion requirement of ≥40 units of blood products, fluconazole prophylaxis was administered (for 5 days or as clinically indicated). When micafungin was used as an antifungal prophylactic agent, 100 mg q24h was administered.

^*b*^ q24h, every 24 hours.

### Study objectives

The primary aim of this study was to determine the rate of *Candida* IP-SSIs during the study period among adult liver transplant recipients according to the peri-transplant antifungal prophylaxis received. Secondary aims included (i) determining the clinical outcomes in each antifungal prophylaxis group and (ii) evaluating the reasons behind antifungal prophylaxis failure in cases of *Candida* IP-SSI occurring in patients who received systemic antifungal prophylaxis.

### Statistical analysis

Continuous variables were calculated as means with standard deviation. Categorical variables were calculated based on frequencies and percentages of the specified group. Comparisons between groups were made with the chi-square test, Fisher exact test, or independent *t*-test as appropriate. A two-sided *P* value of <0.05 was considered statistically significant. *Candida* IP-SSI rates were calculated based on the total number of single liver transplants (denominator) and the total number of *Candida* IP-SSI (numerator) in each prophylaxis group. Statistical analyses were performed using IBM SPSS Statistics (version 29.0; IBM, Armonk, New York).

## RESULTS

**Fig 1:**
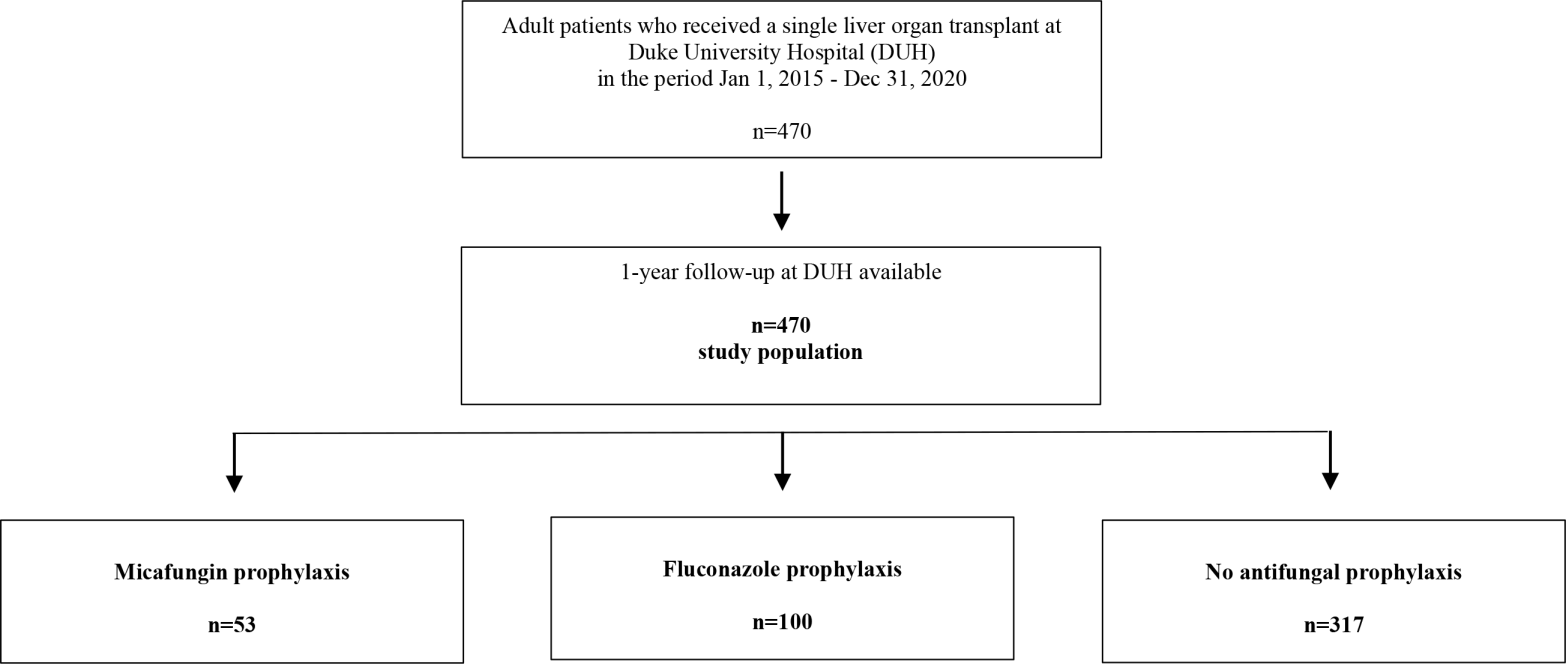
Study population.

Between 1 January 2015 and 31 December 2020, 470 adult single liver transplants were performed at Duke University Hospital (Durham, North Carolina). A 12-month post-transplant clinical follow-up was available for all the patients transplanted, and compliance with institutional prophylaxis recommendations was observed in 96.4% of the cases. Of the 470 adult single liver transplant recipients, 53 (11.3%) received micafungin prophylaxis, 100 (21.3%) fluconazole prophylaxis, and 317 (67.4%) did not receive systemic antifungal prophylaxis in the peri-transplant period (Fig. 1). The duration of antifungal prophylaxis was similar in the fluconazole and micafungin groups (4.1 vs 4.3 days, *P* = 0.56).

### Antifungal prophylaxis groups: baseline characteristics

When comparing the adult single liver transplant recipients who received systemic antifungal prophylaxis with those recipients who did not receive systemic antifungal prophylaxis in the peri-transplant setting, several statistically significant differences were noted (Table 2). This was expected given the targeted antifungal prophylaxis approach in place at our institution during the study period. Patients who did not receive systemic antifungal prophylaxis were older (*P* < 0.01) and had a lower model for end-stage liver disease scores (*P* < 0.01). The underlying disease leading to transplant differed significantly in these study groups (*P* < 0.01). In addition, patients who did not receive systemic antifungal prophylaxis were less likely to be on immunosuppressive therapy (*P* < 0.01) and have end-stage renal disease (*P* < 0.01), ascites (*P* < 0.01), or prior hepatobiliary surgery (*P* = 0.01) in the pre-transplant period. The duration of hospitalization before the transplant was also shorter for patients who did not receive systemic antifungal prophylaxis in the peri-transplant setting (*P* < 0.01). The duration of hospitalization before transplant was also shorter for patients who received fluconazole prophylaxis than for recipients who received micafungin prophylaxis (4.2 vs 8.2 days, *P* < 0.01) (Table 2). The longer pre-transplant hospitalization among those who received micafungin was often due to acute (or chronic) liver failure requiring inpatient management.

**TABLE 2:** Baseline characteristics of adult patients who underwent a single liver transplant at Duke University Hospital in the period between 1 January 2015 and 31 December 2020 stratified by antifungal prophylaxis regimen^*a*^

	Micafungin prophylaxis *n* = 53	Fluconazole prophylaxis *n* = 100	*P*-value (micafungin prophylaxis vs fluconazole prophylaxis)	No antifungal prophylaxis *n* = 317	*P*-value (micafungin and fluconazole prophylaxis vs no prophylaxis)
Male gender, *n* (%)	36 (67.9)	57 (57.0)	0.23	227 (71.6)	0.02
Race, *n* (%)			0.16		0.02
Caucasian	41 (77.4)	72 (72.0)		275 (86.8)	
African American	4 (7.5)	18 (18.0)		27 (8.5)	
Asian	3 (5.7)	3 (3.0)		5 (1.6)	
American Indian or Alaska Native	3 (5.7)	1 (1.0)		2 (0.6)	
Other	0 (0.0)	3 (3.0)		3 (0.9)	
Declined	2 (3.8)	3 (3.0)		5 (1.6)	
Age (years), mean (standard deviation)	52.6 (12.8)	53.7 (10.0)	0.54	57.7 (10.8)	<0.01
BMI, mean (standard deviation)	29.2 (5.0)	30.7 (5.7)	0.11	30.2 (5.5)	0.97
Underlying disease leading to transplant, *n* (%)			0.40		<0.01
Drug-induced acute hepatic necrosis	1 (1.9)	1 (1.0)		1 (0.3)	
Acute hepatic necrosis other	3 (5.7)	4 (4.0)		1 (0.3)	
Cirrhosis biliary (primary and secondary)	3 (5.7)	5 (5.0)		7 (2.2)	
Cirrhosis alcoholic	12 (22.6)	17 (17.0)		55 (17.4)	
Cirrhosis autoimmune	5 (9.4)	4 (4.0)		4 (1.3)	
Cirrhosis cryptogenic	3 (5.7)	2 (2.0)		19 (6.0)	
Cirrhosis NASH	5 (9.4)	24 (24.0)		68 (21.5)	
Cirrhosis HBV related	1 (1.9)	4 (4.0)		2 (0.6)	
Cirrhosis HCV related	6 (11.3)	11 (11.0)		43 (13.6)	
Hepatocellular carcinoma	2 (3.8)	11 (11.0)		79 (24.9)	
Cholangiocarcinoma	0 (0.0)	1 (1.0)		3 (0.9)	
Hepatic epithelioid hemangioendothelioma	1 (1.9)	0 (0.0)		3 (0.9)	
Primary sclerosing cholangitis	6 (11.3)	11 (11.0)		23 (7.3)	
Alpha 1 anti-trypsin deficiency	1 (1.9)	1 (1.9)		5 (1.6)	
Hemochromatosis	0 (0.0)	0 (0.0)		1 (0.3) 3 (0.9)	
Other	4 (7.5)	3 (3.0)			
Pre-transplant immunosuppressive therapy, *n* (%)	10 (18.9)	11 (11.0)	0.22	15 (4.7)	<0.01
Pre-transplant diabetes, *n* (%)	11 (20.8)	31 (31.0)	0.19	110 (34.7)	0.14
Pre-transplant end-stage renal disease, *n* (%)	7 (13.2)	6 (6.0)	0.14	7 (2.2)	<0.01
Antibiotic use in the 4 months prior to transplant, *n* (%)	38 (71.7)	82 (82.0)	0.15	229 (72.2)	0.18
Ascites, *n* (%)	41 (77.4)	70 (70.0)	0.45	182 (57.4)	<0.01
Prior hepatobiliary surgery, *n* (%)	20 (37.7)	36 (36.0)	0.86	80 (25.2)	0.01
Days admitted before transplant, mean (standard deviation)	8.2 (7.5)	4.2 (6.5)	<0.01	1.8 (5.9)	<0.01
MELD score at transplant, mean (standard deviation)	28.3 (9.6)	25.5 (9.8)	0.09	18.1 (7.4)	<0.01
Donor status, *n* (%)			0.56		<0.01
- Brain death	49 (92.5)	87 (87.0)		305 (96.2)	
- Cardiac death	2 (3.8)	8 (8.0)		12 (3.8)	
- Living donor	2 (3.8)	5 (5.0)		0 (0.0)	
Repeat transplantation, *n* (%)	6 (11.3)	4 (4.0)	0.10	2 (0.6)	<0.01
Split liver, *n* (%)	4 (7.5)	12 (12.0)	0.58	2 (0.6)	<0.01
Cold ischemic time (minutes), mean (standard deviation)	326 (126.2)	318 (118.8)	0.72	313.9 (113.7)	0.52
Warm ischemic time (minutes), mean (standard deviation)	37 (6.6)	39 (6.4)	0.12	40 (41.0)	0.53
Primary closure, *n* (%)	48 (90.6)	94 (94.0)	0.52	313 (98.7)	<0.01
Transplant surgery >8 hours, *n* (%)	15 (28.3)	26 (26.0)	0.85	26 (8.2)	<0.01
Roux en Y biliary anastomosis, *n* (%)	17 (32.1)	20 (20.0)	0.11	37 (11.7)	<0.01
Bacterial contamination due to entry into GI tract, *n* (%)	4 (7.5)	1 (1.0)	0.05	0 (0.0)	<0.01
Units PRBC required during surgery, mean (standard deviation)	7.4 (7.1)	7.0 (8.7)	0.79	3.1 (3.5)	<0.01
>6 units PRBC required during surgery, *n* (%)	18 (34.0)	36 (36.0)	0.86	43 (13.6)	<0.01
Anastomotic leak, *n* (%)	6 (11.3)	10 (10.0)	0.79	11 (3.5)	<0.01
Post-transplant renal replacement therapy, *n* (%)	17 (32.1)	25 (25.0)	0.35	17 (5.4)	<0.01
Return to the OR for abdominal surgery within 3 months of transplant, *n* (%)	22 (41.5)	36 (36.0)	0.60	48 (15.1)	<0.01
Duration of antifungal prophylaxis, mean (standard deviation)	4.3 (1.7)	4.1 (1.8)	0.56	−	−
Candida IP-SSI, *n* (%)	5 (9.4)	1 (1.0)	0.02	4 (1.3)	0.09

^*a*^ BMI, body mass index; NASH, non-alcoholic steatohepatitis; HBV, hepatitis B virus; HCV, hepatitis C virus; MELD, model for end-stage liver disease; GI, gastrointestinal; PRBC, packed red blood cells; OR, operating room.

Patients who did not receive systemic antifungal prophylaxis in the peri-transplant setting also had less complex surgical courses with fewer patients in this group requiring operative time above 8 hours (*P* < 0.01), Roux-en-Y biliary anastomosis (*P* < 0.01), extensive use of intra-operative blood products (*P* < 0.01), delayed closure of the abdominal cavity (*P* < 0.01), repeat transplantation (*P* < 0.01), or split liver procedures (*P* < 0.01). Similarly, surgical (peritoneal contamination due to accidental intestinal tract injury or anastomotic leak) and post-surgical (need for renal replacement therapy and abdominal surgery within 3 months of liver transplant) complications were uncommon among patients who did not receive systemic antifungal prophylaxis in the peri-transplant setting when compared to patients who received antifungal prophylaxis (*P* < 0.01). Peritoneal contamination due to accidental intestinal tract injury was more common among patients who received micafungin than patients who received fluconazole (7.5% vs 1.0%, *P* =0.05; Table 2).

### *Candida* IP-SSI rate

During the study period, 10 *Candida* IP-SSIs occurred among 5 of 53 (9.4%) micafungin recipients, 1 of 100 (1.0%) fluconazole recipients, and 4 of 317 (1.3%) recipients who did not receive antifungal prophylaxis. Fourteen *Candida* strains were isolated among the 10 patients with *Candida* IP-SSI (Table 4). *Candida albicans* accounted for 5 (35.7%) of the isolates. Among non-albicans species, *Candida glabrata*, *Candida tropicalis*, and *Candida kefyr* were identified. *Candida glabrata* accounted for 7 (77.7%) of the non-*albicans* isolates. All *C. glabrata* isolates were fluconazole susceptible dose-dependent. Of note, 8 of 10 *Candida* IP-SSI were polymicrobial and were characterized by the concomitant detection of bacterial pathogens and *Candida* (one monomicrobial infection was reported in the micafungin group and one in the no antifungal prophylaxis group).

### *Candida* IP-SSI outcomes

Patients who did not receive systemic antifungal prophylaxis at the time of liver transplant surgery had more favorable clinical outcomes than patients who received antifungal prophylaxis: while no difference in 1-year mortality was observed (*P* = 0.07), their index hospitalization was shorter (*P* < 0.01), and their in-hospital and 30-day mortality rates were lower (*P* = 0.04) (Table 3). Clinical outcomes were similar in the micafungin and fluconazole prophylaxis groups (Table 3).

**TABLE 3:** Outcomes of adult patients who underwent a single liver transplant at Duke University Hospital in the period between 1 January 2015 and 31 December 2020 stratified by antifungal prophylaxis regimen

	Micafungin prophylaxis, *n* = 53	Fluconazole prophylaxis, *n* = 100	*P*-value (micafungin prophylaxis vs fluconazole prophylaxis)	No antifungal prophylaxis, *n* = 317	*P*-value (micafungin and fluconazole prophylaxis vs no prophylaxis)
Length of hospital stay (days), mean (standard deviation)	32.4 (27.5)	22.7 (32.4)	0.07	12.8 (14.8)	**<0.01^*a*^**
In-hospital mortality, *n* (%)	1 (1.9)	3 (3.0)	1.00	1 (0.3)	**0.04**
30-day mortality, *n* (%)	1 (1.9)	4 (4.0)	0.66	2 (0.6)	**0.04**
1-year mortality, *n* (%)	5 (9.4)	7 (7.0)	0.75	11 (3.5)	0.07

^*a*^ Statistically significant values are presented in bold.

### Antifungal prophylaxis failures

Of the 10 *Candida* IP-SSI diagnosed during the study period, 6 occurred in liver transplant recipients who received systemic antifungal prophylaxis in the peri-transplant period and constituted prophylaxis failures (Table 5). Micafungin prophylaxis failure occurred in five patients: two patients received a short duration of prophylaxis (3 days) and developed IP-SSI 6 and 10 days, respectively, after antifungal discontinuation, while three patients developed intra-abdominal candidiasis despite longer courses of prophylaxis, including two that occurred while the drug was still being administered. Also notable, three of the five *Candida* IP-SSIs occurred in the setting of surgical complications (i.e., anastomotic or enteric leaks). Failure of fluconazole prophylaxis was reported in only one patient: in this case, failure occurred on day 3 of prophylaxis with an isolate that was fluconazole susceptible dose-dependent (likely representing microbiologic failure).

## DISCUSSION

This study sheds light on the limitations of antifungal prophylaxis in preventing *Candida* IP-SSI among adult liver transplant recipients at Duke University Hospital over a recent 6-year period (2015–2020).

### Antifungal prophylaxis in adult liver transplant surgery

In line with international recommendations, targeted antifungal prophylaxis was administered at Duke University Hospital in the period of 2015–2020 for adult liver transplant recipients at an increased risk for invasive *Candida* infections (2). Specifically, fulminant liver failure, split liver procedures, and prior *Candida* infections were considered risk factors associated with an increased risk for *Candida* IP-SSI warranting antifungal prophylaxis at the time of surgery. Of note, while re-transplantation, re-operation, renal failure requiring hemodialysis, transfusion of ≥40 units of cellular blood products, and choledochojejunostomy are historically recognized as *Candida* risk factors, their identification did not trigger the deployment of antifungal prophylaxis at our center during the study period (2). Deploying targeted antifungal prophylaxis in the presence of re-transplantation, re-operation, renal failure requiring hemodialysis, transfusion of ≥40 units of cellular blood products, and choledochojejunostomy would have potentially prevented two of the four *Candida* IP-SSIs documented among patients who did not receive systemic antifungal prophylaxis. That said, the low rate (1.3%; 4/317) of *Candida* IP-SSI among patients who did not receive systemic antifungal prophylaxis confirms the overall appropriateness of our targeted strategy, striking the balance between antifungal stewardship and infection prevention.

Of 470 adult single liver transplants performed, 153 (32.6%) were considered at increased risk for invasive *Candida* infections per our protocol and received antifungal prophylaxis. Fluconazole was the recommended antifungal prophylaxis agent and was used in 100 (21.3%) transplant procedures, while micafungin was used in 53 (11.3%). Six *Candida* IP-SSIs occurred among patients who received antifungal prophylaxis. There was a higher rate of *Candida* IP-SSIs (9.4% vs 1.0%, *P* = 0.02) among patients who received micafungin prophylaxis than among those who received fluconazole.

### Possible explanations for the failure of antifungal prophylaxis

Factors influencing the efficacy of antifungal prophylaxis may be related to the pathogen, host, and the drug used.

#### Pathogen-related factors

In the fluconazole group, we documented one prophylaxis failure related to a *C. glabrata* isolate with dose-dependent susceptibility to fluconazole. Isolates in the susceptible dose-dependent category require higher doses of fluconazole to achieve clinical success. Failure in this case was thus likely related to resistance in the isolate and suboptimal fluconazole dosing as prescribed for prophylaxis. While mutations in the *FKS1* and *FKS2* genes that encode the β-1,3-glucan synthase enzyme complex among *Candida* isolates are associated with echinocandin resistance (6, 7), none of the *Candida* isolates identified in the liver transplant recipients who developed a *Candida* IP-SSI in our study population were micafungin resistant based on CLSI breakpoints (Table 4). Thus, echinocandin resistance in the pathogens was not likely the reason for the failure of micafungin prophylaxis.

**TABLE 4:** *Candida* isolates identified among 10 adults who received a single liver transplant at Duke University Hospital in the period between 1 January 2015 and 31 December 2020 and developed a *Candida* spp. IP-SSI^*a*^

Pathogens recovered	Micafungin prophylaxis *n* = 53 patients (five *Candida* IP-SSI cases)	Fluconazole prophylaxis *n* = 100 patients (one *Candida* IP-SSI)	No antifungal prophylaxis *n* = 317 patients (four *Candida* IP-SSIs)	Total *n* = 470 patients (10 Candida IP-SSIs)
*Candida albicans*	2	−	3	5
Fluconazole susceptible dose-dependent	0		0	0
Fluconazole resistant	0		0	0
Micafungin resistant	0		0	0
*Candida glabrata*	3	1	3	7
Fluconazole susceptible dose-dependent	3	1	3	7
Fluconazole resistant	0	0	0	0
Micafungin resistant	0	0	0	0
*Candida tropicalis*	1	−	−	1
Fluconazole susceptible dose-dependent	0			0
Fluconazole resistant	1			1
Micafungin resistant	0			0
*Candida kefyr*	1	−	−	1
Fluconazole susceptible dose-dependent	0			0
Fluconazole resistant	0			0
Micafungin resistant	0			0

^*a*^ Data stratified by antifungal prophylaxis regimen.

#### Host-related factors

Immunosuppression, prolonged antibacterial exposure, and lack of source control are well-recognized factors promoting the failure of antifungal prophylaxis (8). Furthermore, prompt intervention to control sources has been associated with successful clinical outcomes in prior studies on intra-abdominal candidiasis (9, 10). Of the five cases of *Candida* IP-SSI that occurred despite micafungin prophylaxis, three developed in the setting of anastomotic leaks wherein recognition and surgical intervention were not immediate (Table 5). Lack of early intervention may have, therefore, contributed to micafungin failure in these patients. However, it is also worth noting that anastomotic leaks and abdominal surgery within 3 months of liver transplant were equally encountered among patients who received micafungin prophylaxis and patients who received fluconazole prophylaxis, suggesting that prompt intervention and control of leaks were not the sole factor leading to micafungin prophylaxis failure. Other host-related factors may also have been at play. For example, more patients who received micafungin suffered unintentional breaches of the intestinal mucosa during the transplant procedure compared with those who received fluconazole. The relatively small number of patients receiving prophylaxis may have limited our ability to distinguish other important differences between the groups that posed an increased risk for invasive candidiasis.

**TABLE 5:** *Candida* spp. IP-SSIs: clinical details^*a*^

Pathogens (sample)	Antifungal prophylactic drug (dose)	Systemic antifungal indicated per 2015–2020 prophylaxis protocol	Candida IP-SSI risk factors	Candida IP-SSI diagnosis after transplant (days)	Candida IP-SSI clinical presentation	Systemic antifungal prophylaxis at diagnosis of *Candida* IP-SSI	Antifungal prophylaxis before *Candida* IP-SSI diagnosis (days)	Possible explanation for prophylaxis failure
*C. glabrata* (peritoneal fluid)	Micafungin (100 mg)	Yes (micafungin chosen over fluconazole given prior *C. glabrata* infection)	5, 6	13	Candida peritonitis Sepsis	No	3	Premature discontinuation of micafungin (micafungin discontinued on post-transplant day 3)
*C. albicans* *C. glabrata* (peritoneal fluid)	Micafungin (100 mg)	Yes (micafungin chosen over fluconazole given prior *C. glabrata* infection)	5, 6	9	Candida peritonitis secondary to biliary leak Septic shock	No	3	Premature discontinuation of micafungin (micafungin discontinued on post-transplant day 3) and source control (anastomotic bile leak identified on post-transplant day 8)
*C. glabrata* (peritoneal fluid)	Micafungin (100 mg)	Yes (micafungin chosen over fluconazole given prior *C. glabrata* infection)	3, 6	6	Candida peritonitis secondary to biliary leak Asymptomatic, isolated leukocytosis	Yes	6	Source control (anastomotic bile leak identified on post-transplant day 6)
*C. tropicalis* (peritoneal fluid)	Micafungin (100 mg)	Yes (micafungin chosen over fluconazole given prior *C. glabrata* infection)	5, 6	9	Candida peritonitis secondary to enteric leak Asymptomatic, isolated leukocytosis	Yes	9	Source control (enteric leak identified on post-transplant day 9)
*C. albicans* *C. kefyr* (peri-hepatic abscess)	Micafungin (100 mg)	Yes (micafungin chosen over fluconazole given recurrent cholangitis in the pre-transplant period)	1, 3, 5, 7	26	Candida peri-hepatic abscess Asymptomatic, isolated leukocytosis	No	17	Source control (intra-abdominal abscess identified on post-transplant day 26)
*C. glabrata* (blood)	Fluconazole (400 mg)	No (fluconazole used per surgeon’s decision given complicated transplant surgery with Roux anastomosis)	5	3	Candidemia and peritonitis Multifactorial shock (septic and hemorrhagic)	Yes	3	Microbiological failure (*C. glabrata* fluconazole susceptible dose-dependent)
*C. glabrata* (intra-abdominal abscess and blood)	No	No	3	7	Candida intra-abdominal abscess Fever and leukocytosis	−		−
*C. albicans* *C. glabrata* (peri-hepatic abscess and peritoneal fluid)	No	No	5	14	Candida peri-hepatic abscess and peritonitis Asymptomatic, isolated leukocytosis	−		−
*C. albicans* (peritoneal fluid)	No	No	−	13	Candida peritonitis secondary to biliary leak Asymptomatic	−		−
*C. albicans* *C. glabrata* (peritoneal fluid and blood)	No	No	−	4	Candida peritonitis Sepsis	−		−

^*a*^ Candida risk factors: (i) re-transplantation; (ii) re-operation; (iii) renal failure requiring hemodialysis; (iv) transfusion of ≥40 units of cellular blood products; (v) choledochojejunostomy; (vi) *Candida* colonization in the perioperative period; (vii) fulminant liver failure; and (viii) split liver.

#### Antifungal therapy-related factors

In two of the five patients who developed *Candida* IP-SSI despite micafungin prophylaxis, micafungin was discontinued on day 3 post-transplant. The shorter course of antifungal coverage possibly contributed to the development of *Candida* IP-SSI. These cases potentially reinforce the need for a longer duration of antifungal prophylaxis, as suggested by the AST guidelines (2), particularly in patients with surgical complications. Alternatively, one such liver recipient developed a peri-hepatic *Candida* abscess despite 17 days of micafungin prophylaxis. Prior studies also suggest that echinocandin delivery to intra-abdominal sites may be insufficient to achieve concentrations capable of eliminating *Candida* (11–15). Yamada and colleagues analyzed the distribution of micafungin in the ascitic fluid of one patient with an invasive fungal infection and observed that the steady-state concentration of micafungin in the ascitic fluid was only 15% of that in plasma (12). Grau and colleagues performed a population pharmacokinetic study for micafungin in critically ill adult patients with proven or suspected intra-abdominal fungal infection and showed moderate to low penetration of micafungin into the peritoneal fluid with a median area under the concentration-time curve peritoneal fluid-plasma ratio of 0.3 after the first dose and at steady state (13). Zhao and colleagues investigated the tissue spatial and quantitative distribution of micafungin in a murine model and found that at steady state, micafungin diffused into hepatic abscesses at just under 5 μg/g, which was below the reported minimal concentrations that inhibit *Candida* drug-susceptible subpopulations (14). Welte and his team analyzed the concentrations of anidulafungin in the ascitic fluid of seven critically ill patients: anidulafungin concentrations in the ascitic fluid were lower than the simultaneous levels in plasma and below the MIC values for several pathogenic *Candida* strains (15). Taken together, these studies suggest the distribution of micafungin in the abdominal compartment may be limited and concentrations are suboptimal to prevent infection. While two studies have shown similar efficacy of echinocandins and fluconazole in the prevention of invasive fungal infections among high-risk liver transplant recipients (16, 17), Breitkopf and colleagues reported a high incidence (16.0%) of invasive *Candida* infections breaking through echinocandin prophylaxis among adults undergoing first-time liver transplant and receiving echinocandin prophylaxis (micafungin or anidulafungin) for a minimum of 7 days after transplant (18). Finally, no firm conclusions on the efficacy of echinocandin prophylaxis among high-risk liver transplant recipients can be drawn based on the randomized non-inferiority clinical trial performed by Saliba and colleagues wherein liver transplant recipients were randomized to receive prophylaxis with either micafungin or standard of care (caspofungin, fluconazole, or liposomal amphotericin B). While micafungin was determined to be non-inferior to the standard of care in the prevention of invasive fungal infections, the heterogenicity of the standard of care group prohibited definitive conclusions (19).

### Study limitations

This study has multiple limitations. Based on its retrospective design, this study is prone to selection and information bias, and the external validity of this study is hampered by its single-center design. Data generated by this study reflect the epidemiology, outcomes, and management practices associated with *Candida* IP-SSI among adult liver transplant recipients at Duke University Hospital in the period of 2015–2020. Although we observed a statistically significant difference in the rate of *Candida* IP-SSIs among patients who received micafungin prophylaxis compared to patients who received fluconazole prophylaxis, our study was not randomized nor was it powered to adjust for potential confounders. Thus, the association between higher failure rates in the micafungin group compared to the fluconazole group does not provide evidence of superiority. Finally, our study lacks pharmacokinetic data regarding the distribution of micafungin in the intra-abdominal compartment.

### Conclusions

In conclusion, our refined approach to antifungal prophylaxis in the adult liver transplant population successfully identified recipients at low risk for invasive *Candida* infections. Furthermore, our findings highlight the limitations of antifungal prophylaxis in preventing *Candida* IP-SSIs among patients at high risk for candidiasis after liver transplant surgery. We hypothesize that pathogen, host, and drug-associated pharmacokinetic factors contributed to the occurrence of *Candida* IP-SSIs despite antifungal prophylaxis. Given the complexity of liver transplant surgery, our findings reinforce the need for a risk-based, multi-pronged approach to fungal prevention, including targeted antifungal administration in patients with risks for invasive candidiasis in conjunction with close monitoring, especially among patients with complex procedures and surgical complications, with timely intervention/control of surgical leaks. Finally, further investigation into the intraabdominal pharmacokinetics and efficacy of echinocandins for preventing intraabdominal candidiasis following liver transplantation is needed.

## Data Availability

All study data were maintained on a secured REDCap platform offered by the Duke Office of Clinical Research and are available upon request (20).
